# Risk Factors and Surgical Refinements of Postresective Mandibular Reconstruction: A Retrospective Study

**DOI:** 10.1155/2014/893746

**Published:** 2014-08-06

**Authors:** Akiko Sakakibara, Kazunobu Hashikawa, Satoshi Yokoo, Shunsuke Sakakibara, Takahide Komori, Shinya Tahara

**Affiliations:** ^1^Department of Oral and Maxillofacial Surgery, Kobe University Graduate School of Medicine, Kobe 650-0017, Japan; ^2^Department of Plastic Surgery, Kobe University Graduate School of Medicine, Kobe 650-0017, Japan; ^3^Department of Stomatology and Maxillofacial Surgery, Gunma University Graduate School of Medicine, Gunma 371-8511, Japan; ^4^Department of Plastic Surgery, Japanese Red Cross Kobe Hospital, Kobe 651-0073, Japan

## Abstract

*Background.* Postresective mandibular reconstruction is common in cases of oral and mandibular tumors. However, complications such as infection, plate exposure, or plate fracture can occur. We identified several significant risk factors of complications after reconstructive surgery and compared the effectiveness of different surgical techniques for reducing the incidence of complications. *Methods.* This study is a retrospective analysis of 28 oromandibular cancer cases that required reconstructive surgery between January 1999 and December 2011 at Kobe University Graduate School of Medicine in Japan. All cases were classified using Hashikawa's CAT and Eichner's classification methods. Then, we determined whether these classifications and different treatment or surgical methods were significantly related to complications. *Results.* Complications after mandibular reconstruction occurred in 10/28 patients (36%). Specifically, five patients had plate fractures, four had plate exposures, and one had an infection. Radiation therapy and closure without any flaps were significantly related to infection or plate exposure. The wrap-around technique of securing reconstruction plates was used in 14 cases, whereas the run-through technique was used in two cases. *Conclusions.* The success of mandibular reconstruction depends on both mechanical and biological factors, such as the location of defects, presence of occlusions, and the amount of vascularization of the flap.

## 1. Introduction

Surgical resection of oral cavity and mandibular tumors often requires postresective mandibular reconstruction. The goals of mandibular reconstruction are primary wound closure, improvement of phonation and deglutition, and aesthetic restoration of the lower face. There are many techniques for mandibular reconstruction, such as soft-tissue free flaps, reconstruction plates, and bone grafts. Bone reconstruction is often the preferred method. For instance, the fibula free flap technique, which involves resection of vascularized bone from the fibula with a free flap of soft tissue and skin, can be used to reconstruct many types of mandibular defects with relative ease and few complications [1, 2]. However, when the donor bone cannot be harvested or the patient's prognosis is poor, bone reconstruction may not be possible [3, 4]. In such cases, reconstruction plates can be used; however, complications such as infection, plate exposure or fracture, or loosening of the fixation can occur. Minimizing the risk of complications can be challenging, but optimizing the design and fabrication of reconstruction plates and improving surgical techniques may help reduce these risks [5, 6].

**Figure 1:**
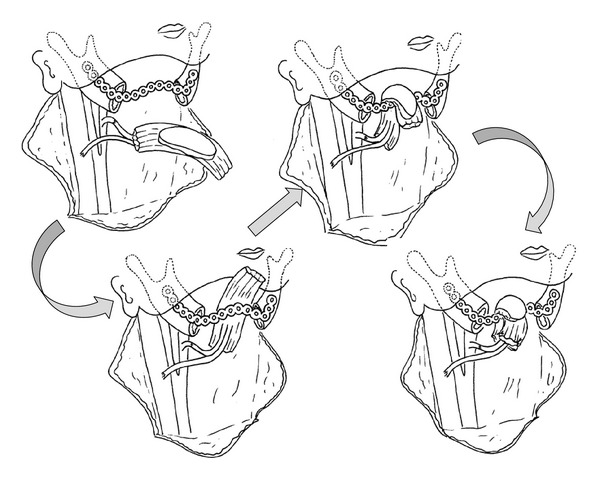
The wrap-around technique involves laying the musculocutaneous flap under the reconstruction plate and then wrapping the plate with muscle, fascia, or a denuded island flap.

**Figure 2:**
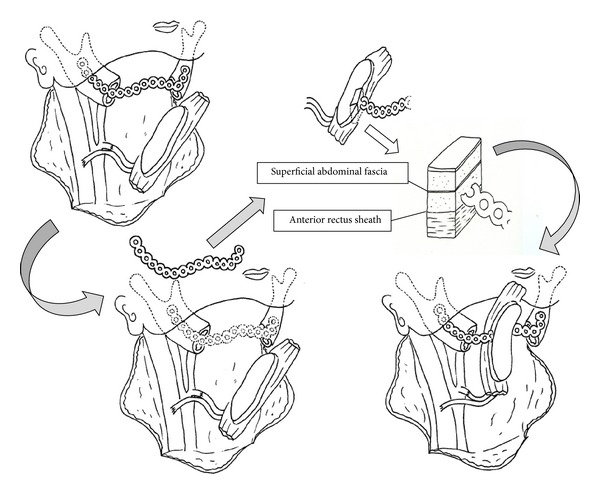
The run-through technique involves inserting the reconstruction plate through a two-island flap. Initially, the plate is positioned for fitting to the mandible but subsequently removed. The plate is penetrated into a layer of deep fascia through the rectus abdominis muscle. Finally, part of the reconstruction plate that is penetrated through the flap is fixed to the mandibular bone. Any surplus flap is denuded and buried under the skin of the neck.

**Figure 3:**
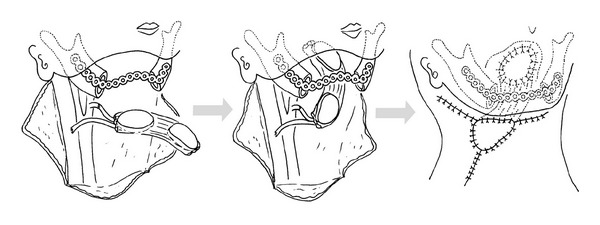
Mandibular reconstruction using a two-island flap. The reconstruction plate is placed on top of the muscle and covered with the skin of the neck.

For example, the risk of plate exposure can be reduced by wrapping flaps around the reconstruction plate to improve its fit and thereby reduce skin tension and dead space. In our institutions, we have adopted the “wrap-around” and “run-through” techniques in mandibular reconstructions performed using rectus abdominis musculocutaneous flaps. The wrap-around technique involves positioning the flap under the reconstruction plate and then wrapping the plate with muscle, fascia, or denuded island flaps (Figure 1) [7]. The run-through technique (Figure 2), which is used in cases where both the skin of the neck and oral mucosa (e.g., the tongue or mandible) are resected, involves inserting the reconstruction plate through a two-island flap (Figure 3) so that the plate is always covered with skin.

In this study, we identified several risk factors of reconstruction complications and compared the effectiveness of several different treatment methods for reducing their impact in patients who require postresective mandibular reconstruction.

## 2. Methods

We performed a retrospective analysis of 28 oromandibular cancer cases that required postresective reconstruction between January 1999 and December 2011 at Kobe University Hospital in Japan. Medical protocols conformed to the Declaration of Helsinki; however, since this was a retrospective study, it was granted an exemption by the local ethics committee.

**Figure 4:**
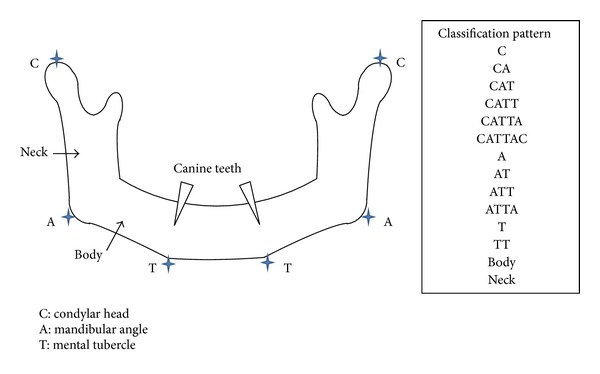
The CAT classification system classifies segmental mandibular defects. “C” refers to defects in the condylar head of the mandible, “A” refers to defects in the mandibular angle, and “T” refers to defects in the mental tubercle. For example, resection of the mandibular angle is classified as “A,” resection of the condylar head and mandibular angle is classified as “CA,” resection of the entire hemimandible is classified as “CAT,” and resection of the mandibular angle and bilateral mental tubercle is classified as “ATT.” In addition, the term “body” is used when only the mandibular body is resected, but the mandibular angle and the mental tubercle are preserved. Similarly, the term “neck” is used when only the mandibular ramus is resected, but the condylar head and the mandibular angle are preserved.

**Figure 5:**
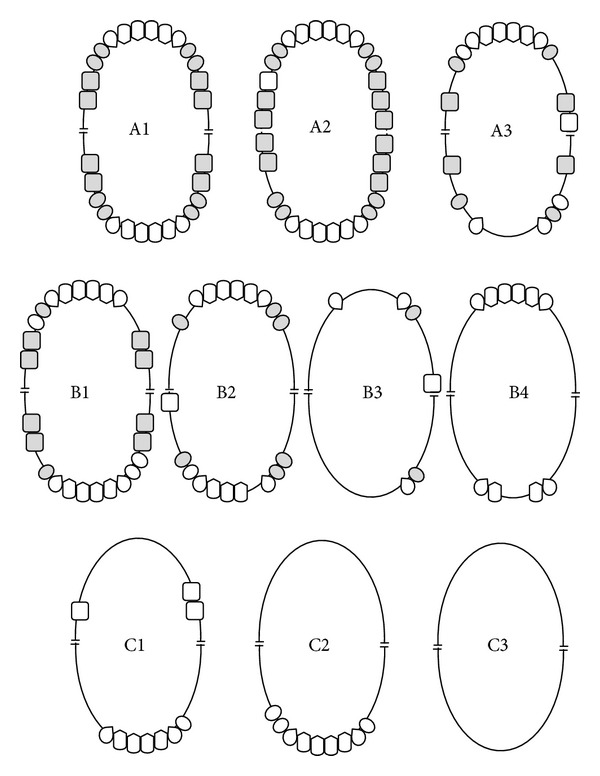
Schematic representation of Eichner's index. Shaded teeth indicate occlusal contacts between natural teeth or fixed prostheses in the premolar and molar regions that constitute occlusal support zones (OSZs). Category A contains 4 OSZs. A1: complete dentition. A2: missing teeth in one arch. A3: missing teeth in both arches. Category B contains 1–3 OSZs or contacts in the anterior area only. B1: 3 OSZs. B2: 2 OSZs. B3: 1 OSZ. B4: contacts in the anterior area only. Category C does not have any OSZs. C1: teeth in both arches. C2: teeth in one arch. C3: edentulous.

For each case, we used two different classification systems, namely, Hashikawa's CAT classification and Eichner's index, to classify segmental mandibular defects and occlusal patterns, respectively. Although the HCL or Urken classification system [8, 9] is a well-known system for classifying mandibular defects, we used the CAT system because it is newer than the HCL system and is more suitable for classifying oncological segmental mandibular defects. In the CAT classification system [10] (Figure 4), “C” refers to defects in the condylar head of the mandible, “A” refers to defects in the mandibular angle, and “T” refers to defects in the mental tubercle. In Eichner's classification system [11] (Figure 5), patients are classified into one of six groups based on the presence or absence of occlusal contacts in the premolar and molar regions. After making these classifications, we examined whether radiotherapy or the type of musculocutaneous flaps used during reconstruction was related to the occurrence of infections or plate exposure. We also compared the effectiveness of the wrap-around and run-through techniques.

We identified statistically significant relationships using the chi-square test. *P* values less than 0.05 were considered statistically significant.

## 3. Results

The patients in this study consisted of 11 women and 17 men with an average age of 70 years (range: 26–89 years). All patients were diagnosed with oral or mandibular squamous cell carcinoma (mandibular mucosa (*n* = 18), oral floor (*n* = 7), tongue (*n* = 2), and buccal mucosa (*n* = 1)) and underwent postresective mandibular reconstruction. Titanium reconstruction plates (Leibinger, Lorenz, or Synthes) were used in all cases.

**Figure 6:**
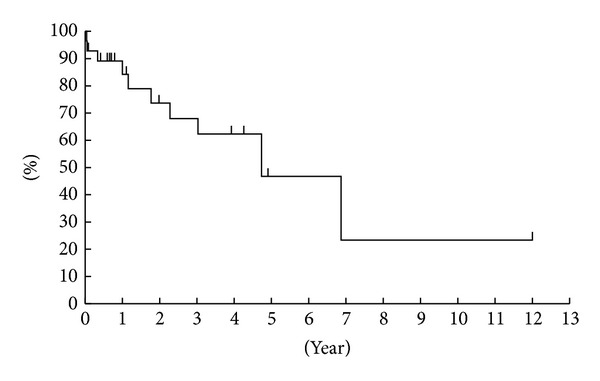
Kaplan-Meier plot showing the success rate of mandibular reconstruction in the patients in this study.

The distribution of cases according to the CAT and Eichner's classification methods is shown in Tables 1 and 2, respectively. In addition, the relationships between different types of treatment and the occurrence of complications are shown in Tables 3–7. Specifically, five patients received radiation therapy (range: 50–70 Gy). Four of these patients received radiation therapy within 12 weeks of surgery, while 1 of the patients had recurred after radiation therapy performed ten years previously (Table 3). Although complications occurred regardless of whether radiation therapy was administered, we found a statistically significant relationship between radiation therapy and complications (Tables 3 and 7). In 27 patients, three different types of soft-tissue free flaps were used, while a simple closure was used in the remaining patient (Table 4). Although at least one patient developed either an infection or plate exposure in each technique, only the simple closure technique was significantly related to complications (Tables 4 and 7). Furthermore, among the 28 cases that we examined, 10 patients (36%) developed complications approximately two years after reconstruction (mean: 25.5 months, range: 0.5–82.4 months; Table 5) and nine patients died within 51 months of surgery. However, a Kaplan-Meier analysis showed that there was no specific period when a complication was likely to occur (Figure 6). Among the 10 patients who developed complications, three received radiation therapy and nine reconstructions were performed with a musculocutaneous flap, but all three types of flaps examined in this study were equally common.

Table 5 also shows the CAT classification and Eichner's index for each case. Complications occurred in two type A patients, four type AT patients, two type T patients, and two type TT patients. Furthermore, among 11 cases with at least one occlusal support zone (e.g., B2 and B3), four patients (36%) had a fractured plate. This complication also occurred in 4/14 (28%) cases that involved a mandibular angle defect. Three of six patients with both occlusal support zones and a mandibular angle defect developed a fractured plate. The other two cases of plate fracture occurred in patients without any occlusions or mandibular angle defects. Chi-square tests showed that only mandibular angle defects and B2 + B3 + B4 occlusions are significantly related to plate fractures (Table 6).

Among the 16 cases that used rectus abdominis musculocutaneous flaps in mandibular reconstructions, the wrap-around technique was used in 14 cases and the run-through technique was used in two cases. Plate exposure only occurred in one patient who had received radiation therapy with the wrap-around technique. No significant relationship was found between plate exposure and the surgical technique used to secure the reconstruction plate (*P* = 0.696).

## 4. Discussion

Our findings suggest that there are three possible causes of complications of mandibular reconstructions, namely, mechanical stress, infection, and radiation therapy. First, several previous studies have reported that bite force and the type of mandibular defect may play a role in plate fractures and detachments. For example, Shibahara et al. reported plate fractures due to mechanical stress in eight of 110 patients who underwent reconstruction after resection of the mandibular angle [12]. Similarly, Boyd et al. [13] suggested that bite force affects both mechanical stresses on reconstruction plates and the success rate of reconstructive surgery. However, not all plate fractures are caused by occlusal stress; fractures may also be caused by excessive intraoperative bending of titanium reconstruction plates [14].

In this study, among the five cases of plate fracture in their study, four cases involved resection of the mandibular angle and had one or two occlusal support zones (B2 and B3). Our findings that mandibular angle defects and B2 + B3 + B4 occlusions are significantly related to plate fractures are consistent with these studies and the hypothesis that these fractures may be due to bite force or mechanical limitations of reconstruction plates.

Second, infections may displace reconstruction plates. For example, in our study, one patient developed a mandibular infection within one month of reconstructive surgery. As a result, the orocervical fistula enlarged, which eventually dislodged the reconstruction plate. Usually, musculocutaneous flaps, which reduce suture tension and dead space, minimize the occurrence of orocervical fistulas that are associated with these problems [15]. However, heavy pedicles or poor blood flow at the tip of the flap can lead to poor outcomes [16]. We believe that this was the case in this patient, who had poor blood circulation due to diabetes.

Third, radiation therapy has several negative effects, including decreased local tissue vascularity and alteration of the bone-to-metal interface, which increases the risk of plate exposure [17]. In addition, previous studies have shown that titanium can cause a backscatter effect, which may increase the risk of local overdoses around the plate and contribute to screw loosening, osteoradionecrosis, and wound breakdown [17, 18]. Our finding that radiation therapy is significantly associated with plate exposure is consistent with these reports. Collectively, our results suggest that reducing the risk of plate fracture will most likely involve reduction of mechanical and biological stresses on the reconstruction plate and surrounding tissues.

Improved surgical techniques may also mitigate the risk of complications after mandibular reconstruction. For instance, in the run-through technique, even if the skin of the neck is weak, the substructure would be the island of the rectus abdominis musculocutaneous flap, and the plate would not be exposed easily. Therefore, we expected that the run-through technique would reduce the risk of complications, but we did not find any significant relationship between plate exposure and the type of flap used. The sample size might be too small to prove these statements statistically at a significant level. Although improved surgical techniques may not reduce the risk of plate exposure, they may have other structural or aesthetic benefits. More research is needed to verify our findings and advance mandibular reconstruction methods.

## 5. Conclusion

Complications of mandibular reconstruction are significantly related to several risk factors, such as the location of mandibular defects, presence of occlusions, and radiation therapy. Improved surgical techniques may enhance the structural integrity or aesthetics of the reconstruction, but they do not seem to reduce the risk of complications.

## Figures and Tables

**Table 1:** Number of cases of plate fracture in each CAT classification. The CAT classification system classifies segmental mandibular defects. “C” refers to defects in the condylar head of the mandible, “A” refers to defects in the mandibular angle, and “T” refers to defects in the mental tubercle.

CAT classification	Patients	Plate fracture cases
	*n* (%)	
A	3 (10.7)	2
TA	10 (35.7)	3
T	5 (17.9)	1
TT	7 (25.0)	0
ATT	1 (3.8)	0
Body	2 (7.1)	0

Total	28 (100)	6

**Table 2:** Number of cases of plate fracture in each Eichner index. Eichner's classification system groups patients according to the presence or absence of occlusal contacts in different dental zones.

Eichner's classification	Patients	Plate fracture cases
	*n* (%)	
A1-3	0	0
B1	0	0
B2	5 (17.9)	2
B3	6 (21.4)	3
B4	1 (3.8)	0
C1	1 (3.8)	0
C2	10 (35.7)	1
C3	5 (17.9)	0

Total	28 (100)	6

**Table 3:** Number of cases with complications in patients who received radiation therapy.

Radiation therapy	Patients *n* (%)	Number of cases with plate infection or exposure
Yes	5 (17.9)	3
No	23 (82.1)	2

Total	28 (100)	5

**Table 4:** Number of cases with complications in each type of soft-tissue free flap.

Type of flap	Patients *n* (%)	Number of cases with plate infection or exposure
No flap	1 (3.8)	1
Radial forearm	4 (14.3)	2
RAM	16 (57.1)	1
PMMC	7 (25.0)	1

Total	28 (100)	5

RAM: rectus abdominis musculocutaneous flap and PMMC: pectoralis major musculocutaneous flap.

**Table 5:** Classification and outcomes of all patients who underwent mandibular reconstruction.

Case number	Age	Sex	Follow-up period (months)	Type of flap	RT	Eichner classification	CAT classification	Complication
1	65	M	13.9	RAM	No	B3	A	Plate fracture
2	64	M	36.3	Radial forearm	No	B2	TA	Plate fracture
3	80	F	82.4	PMMC	No	B2	A	Plate fracture
4	63	M	0.65	PMMC	No	B3	TA	Plate infection
5	61	F	8.5	RAM	No	B2	TA	None
6	54	F	47.1	RAM	No	B2	TA	None
7	68	M	56.8	RAM	No	C2	TA	Plate fracture
8	52	M	12.0	PMMC	No	B3	T	Plate fracture
9	73	F	0.5	None	Yes	C3	TA	Plate exposure
10	70	M	21.2	RAM	Yes	C2	TT	Plate exposure
11	78	M	4.0	Radial forearm	Yes	C2	T	Plate exposure
12	78	F	27.3	Radial forearm	No	C3	TT	Plate exposure
13	26	F	9.5	RAM	Yes	C2	TA	None
14	77	M	7.1	RAM	No	C3	TA	None
15	66	M	7.9	RAM	No	C3	TA	None
16	48	M	2.2	RAM	Yes	C2	ATT	None
17	69	M	43.5	RAM	No	B3	TT	None
18	53	F	47.0	RAM	No	B4	TA	None
19	78	M	48.3	PMMC	No	B3	T	None
20	69	F	50.0	PMMC	No	B3	T	None
21	62	M	5.0	RAM	No	B2	T	None
22	89	F	13.2	PMMC	No	C1	A	None
23	71	M	58.9	RAM	No	C2	TT	None
24	66	F	143.8	RAM	No	C2	TT	None
25	66	M	51.1	RAM	No	C2	TT	None
26	72	F	23.8	Radial forearm	No	C2	Body	None
27	76	M	41.2	RAM	No	C2	TT	None
28	67	F	1.0	PMMC	No	C3	Body	None

RAM: rectus abdominis musculocutaneous flap, PMMC: pectoralis major musculocutaneous flap, and RT: radiation therapy.

**Table 6:** Statistical significance of relationships between plate fracture and CAT and Eichner classifications of patients in this study.

Classification	Chi-square value	*P* value
Eichner's classification		
B2	1.247	0.246
B3	3.702	0.054
B4	0.283	0.595
C1	0.283	0.595
C2	1.207	0.272
C3	1.247	0.246
B2 + B3 + B4	5.100	0.024∗
CAT classification		
A	4.084	0.043∗
TA	0.697	0.410
T	0.007	0.932
TT	2.545	0.111
ATT	0.283	0.595
Body	0.643	0.423

∗Statistically significant (*P* < 0.05,  *χ* ^2^ test).

**Table 7:** Statistical significance of relationships between infection or plate exposure, the type of flap used, and radiation therapy in the mandibular reconstruction of patients in this study.

Risc factor	Chi-square value	*P* value
No flap	4.770	0.029∗
Radial forearm	3.287	0.070
RAM	3.429	0.064
PMMC	0.081	0.776
Radiation therapy	7.370	0.007∗

∗Statistically significant (*P* < 0.05, *χ* ^2^ test).

RAM: rectus abdominis musculocutaneous flap and PMMC: pectoralis major musculocutaneous flap.

## References

[1] Hidalgo DA (1989). Fibular free flap: a new method of mandible reconstruction. *Plastic and Reconstructive Surgery*.

[2] Anthony JP, Rawnsley JD, Benhaim P, Ritter EF, Sadowsky SH, Singer MI (1995). Donor leg morbidity and function after fibula free flap mandible reconstruction. *Plastic and Reconstructive Surgery*.

[3] Boyd JB (1994). Use of reconstruction plates in conjunction with soft-tissue free flaps for oromandibular reconstruction. *Clinics in Plastic Surgery*.

[4] Urken ML, Buchbinder D, Costantino PD (1998). Oromandibular reconstruction using microvascular composite flaps: report of 210 cases. *Archives of Otolaryngology: Head and Neck Surgery*.

[5] Lopez R, Dekeister C, Sleiman Z, Paoli JR (2004). Mandibular reconstruction using the titanium functionally dynamic bridging plate system: a retrospective study of 34 cases. *Journal of Oral and Maxillofacial Surgery*.

[6] Cohen A, Laviv A, Berman P, Nashef R, Abu-Tair J (2009). Mandibular reconstruction using stereolithographic 3-dimensional printing modeling technology. *Oral Surgery, Oral Medicine, Oral Pathology, Oral Radiology and Endodontology*.

[7] Yokoo S, Komori T, Furudoi S (2003). Indications for vascularized free rectus abdominis musculocutaneous flap in oromandibular region in terms of efficiency of anterior rectus sheath. *Microsurgery*.

[8] Jewer DD, Boyd JB, Manktelow RT (1989). Orofacial and mandibular reconstruction with the iliac crest free flap: a review of 60 cases and a new method of classification. *Plastic and Reconstructive Surgery*.

[9] Urken ML, Weinberg H, Vickery C, Buchbinder D, Lawson W, Biller HF (1991). Oromandibular reconstruction using microvascular composite free flaps: report of 71 cases and a new classification scheme for bony, soft-tissue, and neurologic defects. *Archives of Otolaryngology—Head and Neck Surgery*.

[10] Hashikawa K, Yokoo S, Tahara S (2008). Novel classification system for oncological mandibular defect: CAT classification. *Japanese Journal of Head and Neck Cancer*.

[11] Eichner K (1955). A group classification of missing teeth for prosthodontics. *Deutsche Zahnärztliche Zeitschrift*.

[12] Shibahara T, Noma H, Furuya Y, Takaki R (2002). Fracture of mandibular reconstruction plates used after tumor resection. *Journal of Oral and Maxillofacial Surgery*.

[13] Boyd JB, Mulholland RS, Davidson J (1995). The free flap and plate in oromandibular reconstruction: long-term review and indications. *Plastic and Reconstructive Surgery*.

[14] Knoll W, Gaida A, Maurer P (2006). Analysis of mechanical stress in reconstruction plates for bridging mandibular angle defects. *Journal of Cranio-Maxillofacial Surgery*.

[15] Arias-Gallo J, Maremonti P, González-Otero T (2004). Long term results of reconstruction plates in lateral mandibular defects: revision of nine cases. *Auris Nasus Larynx*.

[16] Ijsselstein CB, Hovius SE, ten Have BL (1996). Is the pectoralis myocutaneous flap in intraoral and oropharyngeal reconstruction outdated?. *American Journal of Surgery*.

[17] Ryu JK, Stern RL, Robinson MG (1995). Mandibular reconstruction using a titanium plate: the impact of radiation therapy on plate preservation. *International Journal of Radiation Oncology, Biology, Physics*.

[18] Schöning H, Emshoff R (1998). Primary temporary AO plate reconstruction of the mandible. *Oral Surgery, Oral Medicine, Oral Pathology, Oral Radiology, and Endodontics*.

